# Psychological flow and mental immunity as predictors of job performance for mental health care practitioners during COVID-19

**DOI:** 10.1371/journal.pone.0311909

**Published:** 2024-11-01

**Authors:** Nawal A. Al Eid, Boshra A. Arnout, Thabit A. Al-Qahtani, Neamah D. Farhan, Abeer M. Al Madawi

**Affiliations:** 1 Department of Islamic Studies, College of Hummanities and Social Sciences, Princess Nourah bint Abdulrahman University, Riyadh, Saudi Arabia; 2 Department of Psychology, College of Education, King Khalid University, Abha, Saudi Arabia; 3 Department of Psychology, College of Arts, Zagazig University, Zagazig, Egypt; 4 Department of Learning and Instructor, College of Education, King Khalid University, Abha, Saudi Arabia; 5 College of Islamic Sciences, University of Baghdad, Baghdad, Iraq; 6 Department of Educational Leadership and Policies, College of Education King Khalid University, Abha, Kingdom of Saudi Arabia; The World Islamic Sciences and Education University, JORDAN

## Abstract

**Background:**

Numerous studies indicated that workers in the health sector suffer from work stress, hassles, and mental health problems associated with COVID-19, which negatively affect the completion of their job tasks. These studies pointed out the need to search for mechanisms that enable workers to cope with job stress effectively.

**Objectives:**

This study investigated psychological flow, mental immunity, and job performance levels among the mental health workforce in Saudi Arabia. It also tried to reveal the psychological flow (PF) and mental immunity (MI) predictability of job performance (JP).

**Method:**

A correlational survey design was employed. The study sample consisted of 120 mental health care practitioners (therapists, psychologists, counselors)who lived in Saudi Arabia. Sixty-four were men, 56 were women, and their ages ranged between 27 and 48 (36.32±6.43). The researchers developed three measurements of psychological flow, mental immunity, and job performance. After testing their validity and reliability, these measures were applied to the study participants.

**Results:**

The results found median levels of psychological flow, mental immunity, and job performance among mental health care practitioners. Also, the results revealed that psychological flow and mental immunity were statistically significant predictors of job performance. The psychological flow variable contributed (38.70%) and mental immunity (54.80%) to the variance in job performance of mental health care practitioners.

**Conclusion:**

The current study indicated that psychological flow and mental immunity significantly influenced the job performance of mental health care practitioners. These findings pointed out that human resource management in mental health care institutions in Saudi Arabia must search for procedures that achieve a state of flow and mental immunity for workers to make their jobs more meaningful. Also, these findings indicated the importance of planning interventions to enhance mental health care practitioners’ psychological flow, mental immunity, and job performance to help them cope with work stress effectively and protect them from symptoms of burnout.

## 1. Introduction and literature review

The COVID-19 pandemic has resulted in significant losses, leading to increased employee performance due to many internal and external factors, such as health conditions, stress, and work environment [1]. During the COVID-19 outbreak, the health workforce faced exceptionally high levels of stress and workload, which exposed their mental health to risk, and some of them experienced job burnout. Hence, interventions had to be taken to relieve mental health risks in the long term and to search for mechanisms that would enable them to cope with stress effectively [2].

Positive psychology emerged to focus on studying and enhancing the positive aspects and virtues of humans that increase their ability to face hassles in family and work environments. Psychological flow and mental immunity are vital concepts of modern positive psychology. The study of psychological flow began with Csikszentmihalyi at the end of the twentieth century, when he noticed that people, while practicing their duties, try to engage in the work that they perform with enthusiasm, pleasure, and acceptance, forgetting their interests and other priorities. These persons with a high level of psychological flow spend much time enjoying and being motivated in their work without feeling the passage of time or fatigue [1, 3–5].

Individuals often face many challenges throughout their lives, ranging from daily hardships to major crises, which can lead to the deterioration of mental health. Differences in vulnerability determine the severity of trauma; some persons may experience disorientation in daily tasks, while others may perceive traumatic situations as merely challenging [6]. Workers in certain professions reported energy, knowledge, emotion, and mental capabilities flowing commensurate with the nature of their jobs [7, 8].

Csikszentmihalyi [9] described follow as a valued, memorable, and highly positive experience positioned at an intersection between peak performance and peak experience; the person reaches a state of psychological flow when he performs his work in a distinct and ideal way as he percept it, which transcends the restrictions and challenges they face while controlling the skills that liberate him from dependence and procrastination. For this, we find him trying to accomplish several tasks simultaneously. Flow is a state in which the individual forgets himself, becomes preoccupied with interest and spontaneity as time passes quickly without realizing it, and employs all his abilities. Thus, we can assess the flow state retrospectively using self-report measures. Therefore, psychological flow is considered a pillar of mental health. Thus, the flow state is related to human endeavors and orientations for achievement and excellence to reach the pinnacle of creativity. In various jobs, it was necessary to have psychological flow in those workers to provide services to customers with a high degree of job satisfaction and happiness if they could overcome their internal and external conflicts [5, 9]. The job performance of mental health care practitioners indicates the professionalism with which they carry out the work entrusted to them, as the worker feels fully prepared to provide all the energies and capabilities to achieve the goals [1].

Workers in the health sector suffer from a large number of burdens, which results in burnout and the draining of their energies and capabilities, and this may be reflected in the level of their job performance and the effectiveness of the delivery of health services. Concern for the performance of employees in organizations constitutes a developmental, professional, personal, and social goal that affects their accomplishment of the required level. Workers’ performance contributes to achieving the organization’s goals with high quality, the lowest material cost, and the speed of completion, in addition to the worker’s feeling of job security and well-being [10–12].

Psychological flow allows individuals to develop professionally and enables them to build appropriate psychological capital that motivates them to feel psychological well-being, which increases their productivity more than other individuals who do not have a proper level of psychological flow [13]. The results of previous studies found that there were correlations between the psychological follow among employees (teachers, commercial sector workers, computer programmers, and faculty members) with their emotional stability [14], occupational performance [15], job satisfaction, family harmony [16], optimism [17].

Mental immunity is a construct similar to the biological immune system in some respects; for example, both include independent and reconcilable mechanisms for prevention and self-healing, implicit processes. Similarly, the two systems can be strengthened by training [6]. The resources built into the mental immunity system allow the individual to cope with stresses and hassles [8, 17, 18]. In this way, mental immunity enhances positive behavior and adaptation. Also, mental immunity’s anticipatory and reinforcement mechanisms may increase a person’s well-being [19, 20].

Mental immunity gives individuals the strength to cope with stress, fear, insecurity, inferiority, and viruses like negative thoughts and establish a mental balance. The pentacle model of mental immunity proposed includes five dimensions, mainly self-esteem, adjustment, emotional maturity, and good memories [21]. Self-report measures assess the mental immunity level according to the level of balance among these dimensions, and the higher the level of psycho-immunity, the better mental health. [22]. Thus, mental immunity is a multidimensional construct that provides resistance to psychological trauma. Moreover, mental immunity is known to have a strong relationship with life expectancy, which means that it is associated with phenomena such as well-being. The mental immunity system provides a unifying framework that can better classify cognitive and behavioral effects [23, 24].

Many previous studies [25–29] revealed that mental health care practitioners are exposed to various challenges and stress, which maximized during the outbreak of the COVID-19 epidemic, as they were the first line of defense to help individuals overcome the mental health problems resulting from this pandemic. By reviewing previous studies, it was noted that few studies examined psychological flow and mental immunity among mental health care practitioners, and there are no studies that investigated the psychological flow and mental immunity as predictors of job performance for the mental health workforce, which faced many workloads during the outbreak of the COVID-19 epidemic.

## 2. Objectives

This study sought to investigate psychological flow, mental immunity, and job performance among mental health care practitioners in Saudi Arabia. It also tried to test the possibility of predicting the job performance of mental health care practitioners based on psychological flow and mental immunity. Therefore, this study’s importance is that it reached results that may be useful in planning interventions to enhance mental immunity and psychological flow to improve the job performance of psychological service providers, especially in crises, disasters, and work stress.

### 3. Methodology

#### 3.1. Method and participants

The current study applied the cross-sectional descriptive design to predict the job performance of the psychological services provider’s mental immunity and psychological flow. The study sample size was calculated using the Raosoft Sample size calculator (http://www.raosoft.com/samplesize.html), and the recommended sample size was 106 individuals at the 5% confidence level. The researchers sent an electronic link for study tools via email and WhatsApp messages to a random sample of 145 workers in the field of mental health care (therapists, psychologists, counselors). They asked them to respond after obtaining their consent. The number of respondents who re-sent the study measures were 120 mental health care practitioners who work in Saudi mental health institutions in the Asir region and Riyadh city. Those participants were selected randomly; 64 were men, 56 were women, and their ages ranged between 27 and 48. The study was applied from 7 March 2022 to 28 August 2022 after obtaining approval from the Institutional Ethics Committee of Princess Nourah bint Abdulrahman University (IRB Log Number: 23–0492) and also written informed consent from all participants.

### 3.2. Measurements

#### 3.2.1. Psychological Flow Questionnaire (PFQ) for mental health care practitioners

Due to the lack of psychological flow measures for mental health care practitioners, the researchers developed a tool to measure psychological flow for these workers after reviewing the theoretical literature on psychological flow in general and existing measures specific to other populations such as students. PFQ for mental health care practitioners is a self-report tool prepared by the researchers in this study, consisting of 10 items, which the individual answers using a 5-point Likert scale (1 = strongly disagree to 5 = strongly agree) with a total score ranging from 10 to 50, and a high score indicates a high psychological flow.

#### 3.2.2. Mental Immunity Scale (MIS) for mental health care practitioners

The researchers in this study developed the PIS by reviewing the theoretical literature on mental immunity and existing measures specific to other populations. The MIS consists of 10 items to be answered using a five-point Likert scale (1 = strongly disagree to 5 = strongly agree).

#### 3.2.3. Job performance scale (JPS) for mental health care practitioner

By reviewing the theoretical literature on job performance and existing measures specific to other populations, the researchers developed JPS for mental health care practitioners as a self-report scale consisting of 10 items that the individual answers using a five-point Likert scale (1 = not at all to 5 = incredibly true).

## 4. Data analysis

IBM SPSS 21.0 and JAS 0.18.3.0 software were used to analyze data. Kolmogorov-Smirnov test was applied to test the normality, and the results revealed that the data distribution was normal for psychological flow, mental immunity, and job performance (Kolmogorov-Smirnov Z = 1.261, 1.077, 0.821; Asymp. sig. = 0.083, 0.197, 0.510) respectively. Pearson’s correlation coefficient was used to calculate the correlations between items and the total score of psychological flow, mental immunity, and job performance, as well as Cronbach’s α and McDonald’s ω coefficients and Confirmatory Factor Analysis (CFA) to test the reliability and validity of study measurements. Descriptive statistics, including mean and standard deviation, were also calculated. Regression was also used to examine the predictability of job performance. In this study, the value of statistical significance was 0.05.

## 5. Results

### 5.1. Reliability and validity analysis

#### 5.1.1. The PFQ was applied to 114 Saudi mental health care practitioners to validate its reliability and validity

Principal Component Analysis (PCA) was performed after checking the sample adequacy by the Kaiser-Meyer-Olkin Measure (KMO) test reached (0.70), Bartlett’s test (X^2^ = 230.727, df = 45, p<0.001), and the value of Chi-Square was (87.966, df = 26, p<0.001). Results indicated that the items of the scale were all loaded in one factor called "general psychological flow factor," with a latent root of (5.69), and item loadings ranged between 0.462 to 0.745 (Table 1).

**Table 1:** Correlations and loadings of PFQ items.

Measures	Correlations	Loadings
I set my priorities before I start working.	0.683**	.561
I have specific goals in my work.	0.731**	.522
I make sure to complete my duties quickly and proficiently.	0.489**	.592
I Invest my abilities to achieve my career goals.	0.548**	.664
I enjoy what I do at my job.	0.639**	.462
Time passes slowly, and I do not notice it while doing my job.	0.792**	.580
I feel like I’m doing what others can’t.	0.486**	.745
If I make a mistake in my work, I correct it.	0.803**	.455
I possess skills that enable me to accomplish my duties with minimal effort.	0.648**	.554
I can face any problem at work.	0.747**	.549
Total	-	5.69
% of Variance	-	56.84%

** Statistically Significant at (α≤0.05) level.

**Fig 1:**
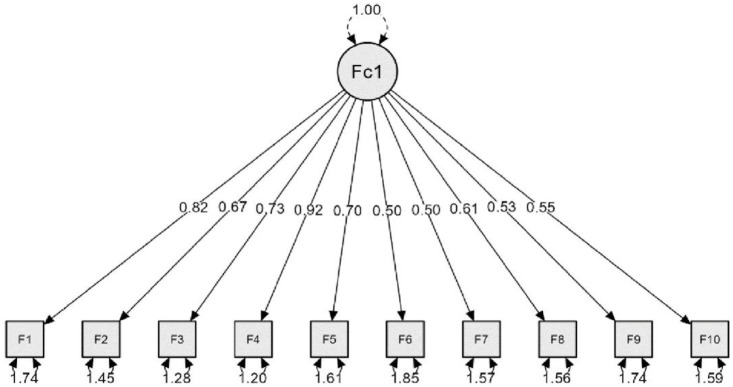
PFQ confirmatory factor analysis results.

Also, the researchers performed PFQ Confirmatory Factor Analysis (CFA), and the results (Fig 1) found that the data gathered from the participants fit with the model of PFQ, with Chi-Square = 101.482, *p* < 0.001, GFI = 0.960, CFI = 0.92, and RMSEA = 0.046.

The researchers also verified the scale’s reliability by calculating Cronbach’s alpha and McDonald’s ω. The value of Cronbach’s α coefficient was (0.870), and McDonald’s ω coefficient was (0.872). These results indicate that the scale has good reliability indicators.

#### 5.1.2. The MIS was applied to 114 Saudi mental health care practitioners to validate its reliability and validity

Principal Component Analysis (PCA) was performed after checking the sample adequacy by the KMO test (0.963), Bartlett’s test (X^2^ = 1679.099, df = 45, p<0.001), and the value of Chi-Square was (84.114, df = 35, p<0.001). The results indicated that the items on the scale were loaded in one factor called "general mental immunity factor," with a latent root of (5.96), and item loadings ranged between (0.413 and 0.716) (Table 2).

**Table 2:** Correlations of items with MIS.

Measures	Correlations	Loadings
What happens to me in my life is of my own making.	0.698**	.578
My personal life is coherent and meaningful.	0.636**	.626
I achieve success one after another.	0.577**	.586
I see the stresses as an opportunity for my personal growth.	0.822**	.716
If I start a job, I persevere until I finish it.	0.752**	.694
I am resourceful and not bored performing my job duties.	0.811**	.638
I am good at developing alternative solutions, even in nervous situations.	0.666**	.562
I can do whatever I need to deal with a problem I’m going through.	0.804**	.413
I find the right person when I need his help	0.733**	.657
I can control my feelings even if others provoke me.	0.825**	.492
Total		5.96
% of Variance		59.62%

** Statistically Significant at (α≤0.05) level

**Fig 2:**
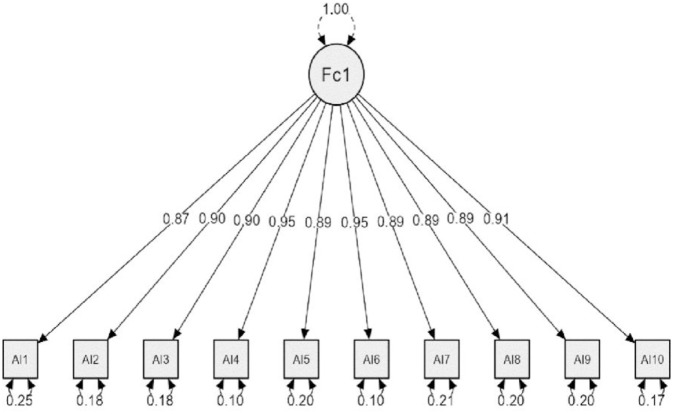
MIS confirmatory factor analysis results.

Also, CFA was conducted for MIS, and the results (Fig 2) found that the data collected from the study sample fit with the model of MIS with Chi-Square = 78.608, df = 35, p < 0.01, GFI = 0.951, CFI = 0.975, RMSEA = 0.068.

Also, the reliability test of MIS by calculating Cronbach’s alpha and McDonald’s ω. The results found that Cronbach’s α coefficient of MIS was (0.931), and McDonald’s ω coefficient was (0.930). These results indicate that the MIS has good reliability indicators.

#### 5.1.3. The JPS was applied to 114 Saudi mental health care practitioners to validate its reliability and validity

Principal Component Analysis (PCA) was performed after checking the sample adequacy by the KMO test (0.772), Bartlett’s test (X^2^ = 211.868, df = 45, p<0.001), and the value of Chi-Square was (91.789, df = 35, p<0.001). The results found that the JPS items were loaded in one factor called "general job performance factor," with a latent root of (6.35), and item loadings ranged between (0.482 to 0.761) (Table 3).

**Table 3:** Correlations of items with JPS.

Measures	Correlations	Loadings
I make a plan to complete assigned tasks on time.	0.435**	.557
My job is my top priority in life.	0.528**	.745
I strive to perform my professional duties accurately and diligently.	0.629**	.691
I sincerely provide services to customers.	0.593**	.629
I collaborate with my co-workers to provide the best services to clients.	0.603**	.516
I enjoy working with and helping reviewers.	0.577**	.571
I try to keep my workload from affecting the quality of my performance.	0.673**	.754
I feel a balance between my work and my personal life.	0.540**	.761
I am careful not to postpone the tasks assigned to me.	0.689**	.482
I organize my tasks at work according to their importance.	0.582**	.641
Total		6.35
% of Variance		63.47%

**Statistically Significant at (α≤0.05) level

**Fig 3:**
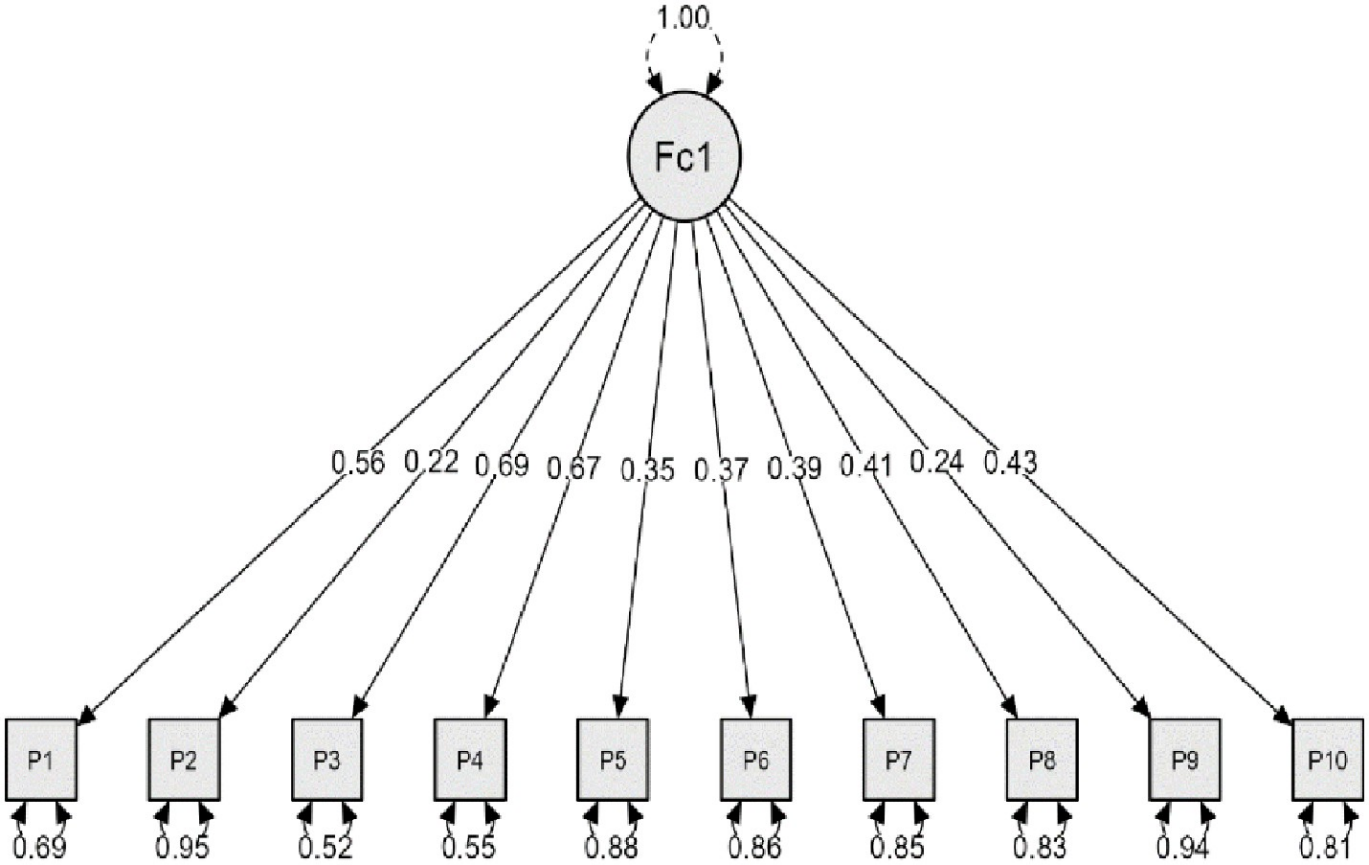
JPS confirmatory factor analysis results.

Also, the researchers performed CFA of JPS, and results (Fig 3) found that the data gathered from the study sample fit with the model of JPS with Chi-Square = 86.210, df = 35, p < 0.01), GFI = 0.978, CFI = 0.91, RMSEA = 0.062.

Also, the reliability test of JPS by calculating Cronbach’s alpha and McDonald’s ω. The results found that Cronbach’s α coefficient of JPS was (0.895), and McDonald’s ω coefficient was (0.896). These results indicate that the JPS has good reliability indicators.

### 5.2. Psychological flow, mental immunity, and job performance among mental health care practitioners

To determine psychological flow, mental immunity, and job performance level among mental health care practitioners, the scores of the study sample were classified into three levels: the low level (from 1 to 2.33), the median level (from 2.34 to 3.67); and the high level (from 3.68 to 5) for each item of the study measures. The total scores were the low level (from 10 to 23.33), the median level (from 23.34 to 36.67), and the high level (from 36.68 to 50).

The means and standard deviations for the level of psychological flow were calculated (29.892 ± 7.643) for the total score. The results showed that the item stipulated "I set my priorities before I start working" has a low level (2.842 ± 1.455), while the item that states: "I can face any problem in my work" has a high level (3.100 ± 1.356). Also, the level of the total score of mental immunity was median (29.450 ± 6.146). The item stated, "What happens to me in my life is of my own making," had a low level (2.808 ± 1.298), while the item that stated: "I can control my feelings even if others provoke me," had a high level (3.275 ± 1.489). Also, the findings found a median level of the total job performance score (30.286 ± 6.992). Also, the item that states, "I make a plan to complete assigned tasks on time" has a low level (2.808 ±1.298), while the item that states: "I organize my tasks at work according to their importance" has a high level (3.479 ±1.288) (Table 4).

**Table 4:** Means and standard deviation of PFQ, MIS, and JPS.

Items	Mean			Standard Deviation			Level		
	PFQ	MIS	JPS	PFQ	MIS	JPS	PFQ	MIS	JPS
1	2.842	2.808	2.798	1.455	1.298	1.356	Median	Median	Median
2	2.925	2.817	2.891	1.348	1.384	1.319	Median	Median	Median
3	2.925	2.833	2.933	1.427	1.324	1.287	Median	Median	Median
4	2.950	2.850	2.975	1.383	1.345	1.279	Median	Median	Median
5	3.008	2.858	2.983	1.435	1.305	1.340	Median	Median	Median
6	3.017	2.917	3.000	1.396	1.464	1.518	Median	Median	Median
7	3.017	2.942	3.008	1.455	1.386	1.324	Median	Median	Median
8	3.033	2.967	3.008	1.561	1.402	1.458	Median	Median	Median
9	3.075	3.183	3.210	1.385	1.378	1.294	Median	Median	Median
10	3.100	3.275	3.4790	1.356	1.489	1.288	Median	Median	Median
Total	29.892	29.450	30.286	7.643	6.146	6.992	Median	Median	Median

### 5.3. The predictive ability of psychological flow and mental immunity with the job performance of mental health care practitioners

The linear regression results in Table 5, with R2 = 0.882, indicate that psychological flow and mental immunity variables can explain 88.20% of the job performance variance. The ANOVA of the regression model proved to be statistically significant (F = 204.759, *p* < .000) and revealed that psychological flow might influence the changes in job performance with a regression coefficient of β = 0.548 (t = 5.195, *p* <0.01) and mental immunity variable might influence of the changes in the job performance with a regression coefficient of β = 0.387 (t = 3.473, *p* <0.01). The findings in Table 5 illustrated that psychological flow and mental immunity variables might significantly positively affect job performance.

**Table 5:** Results of regression analysis of psychological follow and mental immunity on job performance.

Model	R^2^	Durbin-Watson	F	Independent Variables	Unstandardized Coefficients		Standardized Coefficient	t	Sig.
					B	SE	Beta		
1	0.882	1.848	204.759	Constant	2.504	1.507		1.662	0.099
				Psychological flow	0.548	0.105	0.541	5.195	0.000
				Mental immunity	0.387	0.111	0.361	3.473	0.001

* Dependent Variable: Job performance

* Statistically Significant at (α≤0.05) level

## 6. Discussion

The current study found median levels of mental health care practitioners’ psychological flow, mental immunity, and job performance. These results are consistent with the findings of the Al-Sawafi study [1], which found median levels of psychological flow. The workers in the field of providing mental health services are exposed to multiple situations that cause a lot of stress, especially professional ones. Also, the results revealed that psychological follow and mental immunity statistically affect job performance. The flow in the work setting prevents workers from burnout and a sense of inefficiency because the flow is a delightful performance state; it was found to be positively related to job performance [30–33]. This is consistent with what was mentioned: the flow experience is intense participation and intense interest from the individual in the activity he performs [34]. For this reason, it has a positive association with psychological capital (self-efficacy, hope, flexibility, optimism) and reduces fatigue[35–37]. Flow that depends on balancing challenges and skills achieves positive results for the worker and the organization [37–40].

Increasing workloads reduce workers’ well-being and threaten mental health [31–33]. Hence, human resource management in organizations must search for mechanisms that achieve a state of flow among employees, making them realize that their work is meaningful and enjoyable, regardless of the burdens and challenges of job tasks [31, 32].

Many studies have found that the stress that workers are exposed to in the work environment not only affects their health and psychological condition but also negatively affects the individual’s level of work performance, and thus, the individual’s level of competence decreases [33, 35]. Workers in the mental health care sector faced stress that increased with the spread of the COVID-19 epidemic, which reduced their ability to cope with this stress and frustration. Then, their psychological flow, mental immunity, and job performance reached a median level. As a result of the continuous job stress, their ability to control their emotions and resist daily frustrations at work is weakened [31, 33–35].

The levels of psychological flow, mental immunity, and job performance were median because mental healthcare practitioners suffer from high exposure to stressful events and mental health problems resulting from them, including depression, anxiety, post-traumatic stress disorder, sleep loss, and troubled family relationships [35, 37] studies have shown that job dissatisfaction and work stress are the most common problems among mental health care practitioners in many countries. Therefore, it is increasingly recognized that work stress is one of the causes of occupational health risks that reduce workers’ satisfaction and productivity and increase their absenteeism, work turnover, and lack of job performance [41–43]. There is no doubt that the persistence of these stress and the worker’s lack of personal resources that enable him to face these stress effectively leads to the erosion of values, reduces the worker’s spirituality and will, dissipates his personality, decreases his productivity, and low achievement, all of which represent symptoms of job burnout, and thus this causes a decrease in the level of flow. Psychological flow and mental immunity result in a reduction in job performance [41, 43].

Also, the results revealed that psychological follow and mental immunity statistically affect job performance. The flow in the work setting prevents workers from burnout and a sense of inefficiency because the flow is a delightful performance state; it was found to be positively associated with occupational performance [30–32, 34]. This is consistent with what was mentioned: the flow experience is intense participation and interest from the individual in the activity he performs [34]. For this reason, it has a positive relationship with psychological capital (self-efficacy, hope, flexibility, optimism) and reduces fatigue [35–37]. Flow that depends on balancing challenges and skills achieves positive results for the worker and the organization [37–40].

Mental immunity empowers the worker to perform their job tasks without feelings of stress. Mental immunity is a set of personal characteristics that make the individual able to withstand the effects of exhaustion and pressure, integrate the acquired experience, and protect the individual from harmful environmental influences [33, 35, 40, 41]. The study by Abdel Nasser [33] found a significant correlation between mental immunity, occupational stress, and the professional competence of workers. The psychological immune system is one of the personality factors responsible for coping with stress and psychological fatigue to achieve mental health [6]. Gilbert et al. [36] also added that mental immunity is used to reduce the number of biases and cognitive mechanisms that protect the individual from extreme negative feelings by ignoring and constructing information to make the individual’s current situation more likely to agree and have more helpful alternatives. Therefore, mental immunity is one of the most critical mental variables that help workers cope with work stress and terrible events and raise professional competence [42, 43].

Lack of mental immunity in the individual causes a loss of self-control, surrender to failure, isolation, loss of a sense of pleasure in life, a defect in the criteria for judging things and situations, closure and intellectual stagnation, weak emotional maturity, and a decrease in the individual’s performance [8]. The results of Dubey and Shahi’s [35] study showed that those with high mental immunity are less affected by the stress and occupational burnout of workers in the medical professions. Abdel Nasser’s study [33] also found that the higher the mental immunity, the higher the level of professional competence for workers. Hanafi’s [39] study found a relationship between mental immunity and social competence. Also, Stankovic et al. [40] found that mental immunity predicted burnout syndrome.

We can conclude that the current findings agree with previous studies showing a negative impact of the COVID-19 outbreak on workers’ job performance, well-being, and mental health [44–48].

## 7. Limitations and future directions

Despite these vital findings about the possibility of predicting the job performance of mental health care practitioners through psychological flow and mental immunity, this study has several limitations. One of these limitations is that the current research is a descriptive and predictive design and was not intrusive in revealing the effect of developing psychological flow and mental immunity on the job performance level of mental health care practitioners. Also, this study is quantitative and not qualitative or mixed design. Hence, future interventional studies can be conducted on enhancing the psychological flow and mental immunity on the job performance level, and qualitative and mixed studies examining psychological flow and mental immunity in mental health care practitioners can be conducted extensively.

## 8. Conclusion

This correlational study investigated the level of psychological flow, mental, and job performance. It detected the predictability of job performance by psychological flow and mental immunity among mental health care practitioners in Saudi Arabia. The study found median levels of psychological flow, mental immunity, and job performance. Also, the study revealed that psychological flow and mental immunity were significant statistical predictors of job performance among mental health care practitioners. These results pointed out that human resource management in mental health care institutions in Saudi Arabia must search for procedures that achieve a state of flow and mental immunity among mental health care practitioners to make their work more meaningful and enjoyable. Also, these findings indicated the importance of planning interventions to enhance mental health care practitioners’ psychological flow, mental immunity, and job performance to help them cope with work stress effectively and protect them from symptoms of burnout.
